# The Role of MAPK and Dopaminergic Synapse Signaling Pathways in Antidepressant Effect of Electroacupuncture Pretreatment in Chronic Restraint Stress Rats

**DOI:** 10.1155/2017/2357653

**Published:** 2017-10-17

**Authors:** Xinjing Yang, Zhuo Guo, Jun Lu, Bingcong Zhao, Yutong Fei, Jing Li, Huili Jiang, Lan Sun, Yu Wang, Yang Sun, Tuya Bao

**Affiliations:** ^1^School of Acupuncture-Moxibustion and Tuina, Beijing University of Chinese Medicine, Beijing 100029, China; ^2^School of Chinese Integrative Medicine, Hebei Medical University, Shijiazhuang 050091, China; ^3^Centre for Evidence-Based Chinese Medicine, Beijing University of Chinese Medicine, Beijing 100029, China; ^4^School of Psychological and Cognitive Sciences, Peking University, Beijing 100871, China

## Abstract

Acupuncture has demonstrated the function in ameliorating depressive-like behaviors via modulating PKA/CREB signaling pathway. To further confirm the antidepressant mechanism of EA on the mitogen-activated protein kinase (MAPK) and dopaminergic synapse signaling pathways, 4 target proteins were detected based on our previous iTRAQ analysis. Rats were randomly divided into control group, model group, and electroacupuncture (EA) group. Except for the control group, all rats were subjected to 28 days of chronic restraint stress (CRS) protocols to induce depression. In the EA group, EA pretreatment at Baihui (GV20) and Yintang (GV29) was performed daily (1 mA, 2 Hz, discontinuous wave, 20 minutes) prior to restraint. The antidepressant-like effect of EA was measured by body weight and open-field test. The protein levels of DAT, Th, Mapt, and Prkc in the hippocampus were examined by using Western blot. The results showed EA could ameliorate the depression-like behaviors and regulate the expression levels of Prkc and Mapt in CRS rats. The effect of EA on DAT and Th expression was minimal. These findings implied that EA pretreatment could alleviate depression through modulating MAPK signaling pathway. The role of EA on dopaminergic synapse signaling pathways needs to be further explored.

## 1. Introduction

Depression is a complex mood disorder characterized by both emotional and cognitive symptoms, and it makes a significant contributor to the global burden of disease [1, 2]. Unfortunately, little is still known about the etiology and pathophysiology of depression [3]. Antidepressant drugs have been applied as mainstream treatment for depression over the past decades [4]. However, approximately 60% patients do not respond well to antidepressant drugs [5]. Four kinds of common antidepressant drugs, including selective 5-serotonin reuptake inhibitors (SSRIs), serotonin-noradrenergic reuptake inhibitors (SNRIs), tricyclic antidepressants (TCAs), and monoamine oxidase inhibitors (MAOIs), have displayed potential high relapse rates and unpredicted adverse reactions [6–8]. Suggesting that the treatment of depression has other unknown treatment targets needs to be explored. Researchers are focusing studies on searching alternative therapies as compensation for lack of effective treatment depression [9, 10].

As a traditional Chinese medical therapy, acupuncture has been proved to be effective and safe in the treatment of depression [11–13]. However, the underlying mechanism of acupuncture on depression is still unclear. Most studies of EA for depression focused on some individual indicators of chronic stress-related hypothalamus-pituitary-adrenal (HPA) axis, inflammation, serotonin system, or dopaminergic system [14, 15]. Few studies have observed the overall regulation effect of EA on depression.

The hippocampus, which is part of the limbic system and develops nerve fiber connectivity with emotion-related brain regions, is the most important and commonly studied brain region in depression research. Stress can impact hippocampal plasticity in many ways [16]. Study has indicated that EA treatment could act on depression by modulating key factors such as HPA axis and serotonin system in the hippocampus [17]. Our previous researches revealed that acupuncture could ameliorate depressive-like behaviors in depression model rats through regulating PKA/CREB signaling pathway in the hippocampus [18].

MAPK signaling pathway, especially Classical MAP kinase pathway, is the main findings of our previous study in EA for depression. In recent proteomics study, based on proteomics and bioinformatics analysis, we found EA improved depressive-like symptoms through regulating differential proteins [19]. Noticeably and importantly, protein kinase C (Prkc) and the microtubule-associated protein Tau (Mapt) in the MAPK signaling pathway are among the twenty-seven differential proteins and not involved in our previous MAPK signaling pathway studies. Prkc has been found to play an important role in patients with psychiatric disorders and also in the therapeutic mechanism of psychoactive drugs [20]. Mapt is one of the major neuronal microtubule-associated proteins (MAPs), exhibiting its highest expression over frontotemporal neocortex and hippocampus in the mammalian brain [21]. The dopamine transporter (Slc6a3, DAT) and tyrosine hydroxylase (Th) involved in the dopaminergic synapse signaling pathway are two novel indicators involved in differential proteins we screened. Monoamine hypothesis is a major topic in depression research. However, less studies focus on the role of EA on dopaminergic synapse signaling pathway. The four proteins were not being concerned and still not validated in our previous research. Therefore, we detected Prkc and Mapt in the present study to further confirm and clarify the regulation effect of EA on MAPK signaling pathway in CRS rats. In the meantime, we chose DAT and TH as target proteins to further study the relationship of dopaminergic synapse signaling pathway and the antidepressant effect of EA.

## 2. Methods

### 2.1. Animals and Groups

Male specific pathogen-free (SPF) Sprague-Dawley (SD) rats, aged 5 weeks, were obtained from Charles River Laboratories of Beijing, China (license number SCXK (Jing) 2012-0001). All rats were housed 3 days for environment acclimatization (temperature 18–26°C, humidity 55%, in 12-hour light/dark cycles (light on at 8:00 a.m.) with free access to food and water). All surgery was performed under chloral hydrate anesthesia, and all efforts were made to minimize animal suffering and to be consistent with the 3R principle of reduction, replacement, and refinement. The experimental procedures were conducted in compliance with the Guidance Suggestions for the Care and Use of Laboratory Animal, issued by the Ministry of Science and Technology of China [22], and received approval from the Animal Ethics Committee of Beijing University of Chinese Medicine (permission number Kj-dw-18-20131012).

### 2.2. Experiment Design

45 rats were randomly divided into three groups: control group, model group, and EA group (15 rats in each group). In the control group, rats were group reared in three cages, 5 rats in each cage. They were deprived of food and water for 6 hours (9 a.m. to 3 p.m.) every day, for 28 days. No model induction and treatment were performed. In the model group, each rat was socially isolated by placing each rat in a separate cage and underwent CRS procedure. In the EA group, EA pretreatment was conducted daily before the restraint, which was the same as the procedure in the model group.

### 2.3. Animal Model Establishment

The chronic restraint stress (CRS) model was established in this study. CRS induces depression-like behaviors accompanied by physiological changes in rats [23–25]. Therefore, CRS could be built as an appropriate instrument to mimic clinically relevant symptoms and characteristic in depression [26]. Social isolation and chronic restraint stress were conducted daily for 28 consecutive days by the following method: rats were restrained in a self-made cylinder-shaped wire net (20 cm length and 5 cm diameter) for 6 h from 9 a.m. to 3 p.m., during the rats' normal low-activity period. After restraint, they were released with free access to water and food.

### 2.4. EA Pretreatment

During EA administration, rats were maintained within a cloth bag. Baihui (GV20) and Yintang (GV29) were selected for EA pretreatment. GV20 is located above the apex auriculate, on the midline of the head, and GV29 at the midpoint between the two eyes [27]. Sterilized disposable stainless steel needles (0.2 × 25.0 mm, Huan Qiu Brand, manufactured by Suzhou Medicine Co., Ltd., Suzhou, China) were inserted obliquely as deep as 5 mm for both points. Following the insertions, electrodes were added to the handle of needles (EA apparatus used: SDZ-V electronic needle therapy instrument, manufactured by Suzhou Medical Supplies Co., Ltd.). EA pretreatment commenced 30 minutes before CRS procedure, 20 minutes per session, and 1 session daily for 28 days. Electricity simulation parameters were 1 mA, 2 Hz, and discontinuous wave.

### 2.5. Body Weight

All rat body weights were measured on day 0, day 7, day 14, day 21, and day 28 of the experiment.

### 2.6. Open-Field Test (OFT)

The OFT is commonly used to measure general locomotor activity and willingness to explore in rodents [28].

The OFT was conducted at day 0, day 7, day 14, day 21, and day 28 of the study as previously described with modifications [29]. A wood apparatus, composed of a square arena (80 × 80 cm^2^) with a 40 cm high wall, was divided into 25 × 25 cm^2^ equal squares which had been drawn in the floor of the arena. A single rat was gently placed in the center of the floor and allowed to familiarize itself with the apparatus for 3 minutes, and then a camera installed 1 meter away from the apparatus recorded the activities of the rat. The crossing times (defined as at least three paws in a square) and the rearing times in 3 minutes (defined as the rat standing upright on its hind legs) were monitored and counted by two observers who were blind to the experimental design. After one rat finished the test, alcohol was applied to clean the whole apparatus to exclude the intervention of odor signals.

### 2.7. Sample Collection

The hippocampus was carefully dissected out from 12 Sprague-Dawley rats on day 28. Rats were deeply anesthetized with 10% chloral hydrate (0.3 mL/100 g, i.p.) and then (1) cut along the coronal suture and sagittal suture and pull off both sides of parietal bone and inter parietal bone, (2) open the skull, remove the cerebral cortex covering it, and separate the rest of the hippocampus from the cortex covering it along the surface of the hippocampus towards the ventral part of the hippocampus, and (3) free the hippocampus from the surrounding tissue, and the hippocampus was isolated and stored at −80°C for further analysis.

### 2.8. Western Blot

Hippocampal tissue was immediately sonicated in RIPA buffer with protease inhibitors PMSF. The samples were centrifuged at 12,000 rpm during 10 minutes at 4°C and protein-containing supernatants were collected in tubes kept on ice for protein analyses. Protein (quantified by BCA assay, BCA protein assay kit, Dingguo, Beijing, China) for each lane was boiled at 95°C for 5 min and electrophoresed on SDS-polyacrylamide gels (GenView) and then transferred to polyvinylidene fluoride (PVDF) membranes (PALL). The membranes were blocked in 5% milk in Tris-buffered saline with Tween (TBST) for 1 h at room temperature (RT) and incubated at 4°C overnight with the following primary antibodies: Anti-Dopamine Transporter (catalogue number ab5990), Anti-Tyrosine Hydroxylase (catalogue number ab75875), Anti-PKC delta (catalogue number ab47473), and Anti-MAP2 (catalogue number ab5392) (1 : 1000 in 5% BSA-TBST). Horseradish peroxidase-conjugated secondary antibodies and an ECL (PIERCE) kit were used to detect protein signals. Selected films were quantified using Quantity One software and are shown as density relative to *β*-actin.

### 2.9. Statistical Method

Data were expressed as mean ± SD. Statistical analysis of data was carried out by one-way analysis of variance (ANOVA) after the test of normal distribution and homogeneity of variance, followed by the LSD post hoc test. Kruskal-Wallis* H* test was used for abnormal distribution. A value of *p* < 0.05 was considered to be statistically significant.

## 3. Results

### 3.1. Effects of CRS and EA Pretreatment on Body Weight

**Figure 1:**
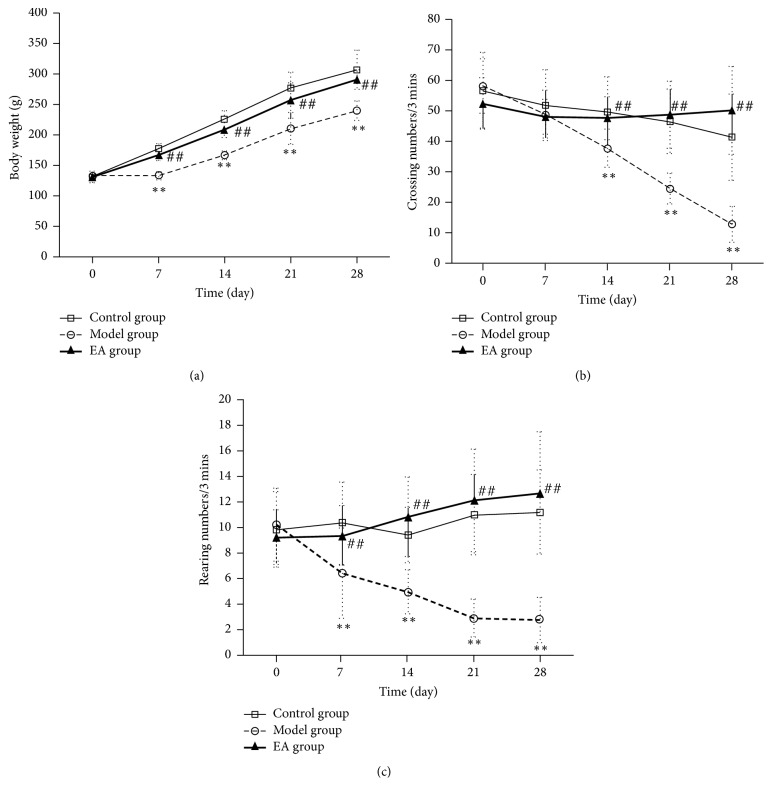
Effects of CRS and EA pretreatment on body weight and locomotor activity in the open-field test before and after treatment ($\overset{-}{x}$  ± SD, *n* = 15). (a) Body weight (g). (b) and (c) Open-field test. ^*∗∗*^*p* < 0.01 as compared with control group; ^##^*p* < 0.01 as compared with model group.

As shown in Figure 1(a), there was no significant difference on body weight among the three groups at the beginning of the study (*p* > 0.05). At the end of the study (day 28), compared with the control group and the EA group, the model group showed the body weight was significantly decreased (*p* < 0.01).

### 3.2. Effects of CRS and EA Pretreatment on OFT

The results of OFT revealed no significant difference among the three groups in crossing (*p* > 0.05) and rearing (*p* > 0.05) at the beginning of the study. At the end of the study (day 28), the results indicated that CRS exposure significantly decreased crossing and rearing numbers as compared to control rats and EA pretreatment rats (*p* < 0.01). In addition, EA pretreatment increased locomotor activity (crossing) and exploration willingness (rearing), and the differences were statistically significant in comparison with the model group (*p* < 0.01) (Figures 1(b) and 1(c)).

### 3.3. Effects of CRS and EA Pretreatment on Candidate Proteins Expression (See Figures 2 and 3)

#### 3.3.1. Prkc Expression

Prkc expression was significantly increased in the hippocampus after CRS exposure compared with the control group (*p* < 0.01). And 28-day EA pretreatment reduced Prkc expression compared with the model group (*p* < 0.05).

#### 3.3.2. Mapt Expression

Conversely, CRS significantly decreased Mapt levels in the hippocampus compared with the control group (*p* < 0.05), while EA pretreatment increased the Mapt expression compared with the model group (*p* < 0.05). In the meantime, Mapt expression in the hippocampus reverted to the normal level after EA pretreatment (compared with the control group, *p* > 0.05).

#### 3.3.3. DAT Expression

On the day of 28, DAT expression in the hippocampus was significantly increased in the model group compared with the control group (*p* < 0.01). After EA intervention, DAT showed lower expression compared with model animals, while this difference did not reach statistical significance (*p* > 0.05). And the DAT expression in the EA group was higher than that in the control group (*p* < 0.05).

#### 3.3.4. Th Expression

In the hippocampus, compared with the control group, CRS reduced Th expression in the model group. EA increased Th expression compared with the model group, while the differences were not significant (*p* > 0.05).

## 4. Discussions

In the present study, the CRS model has been built, and the Western bolt has been used to validate the previous iTRAQ analysis results. In recent years, iTRAQ that is combined with LC-MS/MS analysis has been used as a powerful quantitative proteomic method. This method is more sensitive than the traditional proteomic approaches, especially for the quantifying low-abundance proteins in the tested samples [30, 31]. According to the protein quantitative verification result of Western blot, we found that the expressions of Prkc and Mapt are consistent with the previous iTRAQ analysis result.

Prkc plays an important role in regulating neuronal excitability, neurotransmitter release, and long-term alterations in gene expression and plasticity [32]. A study has reported that the expression and the function of Prkc are altered in animal models with depression [33]. Prkc is also a key component of the phosphoinositide (PI) signaling system related to serotonin (5-hydroxytryptamine, 5-HT), which has been found to have close relationship with depression [34]. Prkc*δ* may be important in ethanol-dependent neuronal hyperexcitability [35]. After inducing of CRS, we can find the Prkc expression increase in the hippocampus. It is consistent with the finding of Coull et al., who found a significantly higher number of [^3^H] phorbol dibutyrate that had been used as a marker of Prkc in frontal cortex [36]. The treatment of rats with antidepressants could cause decreased Prkc activity in the brain [37]. Clinical research showed the same tendency: one study observed increased the formation of Prkc in platelets of depressed patients [38]. This evidence suggested that the increased Prkc might be associated with the pathophysiology of depression. In the present study, the Western blot result was consistent with the iTRAQ, which showed EA could decrease the expression of Prkc in the hippocampus.

Suggesting Mapt expression is increased in response to nerve growth factor, most of researches on Mapt are mainly referred to Parkinson disease or Alzheimer's disease. Tau-Induced Defects will influence synaptic plasticity, learning, and memory and are essential for long-term depression in the hippocampus [39, 40]. Although the role of Mapt in depression has not been explored clearly to date, there is still evidence that Mapt is presented in the postsynaptic compartment of many neurons [41]. Mapt knockout in mice leads to defects in hippocampal long-term depression, as well as mild network-level alterations in brain function [42]. A meta-analysis of genome-wide analysis of over 106000 individuals identifies 9 neuroticism-associated loci associated with neuroticism and included candidate genes on chromosome 17 Mapt [43]. These may provide indirect evidence that Mapt is associated with depression. In the present study, both the Western blot result and the iTRAQ result showed that the expression of Mapt in the hippocampus was reduced in depressive status but was increased significantly after EA pretreatment.

The pathway analysis could reveal the most important biochemical metabolic pathways and signal transduction pathways involved in differential proteins. It shows that Mapt and Prkc are important proteins in the MAPK signaling pathway. MAPK signaling is an intracellular process involved in the initiation of cellular processes, such as differentiation and proliferation [44]. The pathway is activated by stress [45]. A rapid downregulation of Prkc*δ* can activate MAPK [46]. Prkc plays a critical role in Ca2+ dependent Erk signaling with biochemical requirements [47]. Furthermore, Manosso et al. demonstrated that the activation of the Prkc-mediated pathway is associated with the antidepressant-like behaviors [48]. Our previous studies have proved that acupuncture had a significant antidepressant-like effect on chronic unpredictable mild stress- (CUMS-) induced depression model rats by regulating the ERK signaling pathway in the hippocampus [29, 49]. Another study has found that EA could act on depression through enhancing p-ERK1/2 and p-p38 in the hippocampus [50]. In terms of DAG-PKC-ERK involved in MAPK signaling pathway, we speculated that EA may inhibit Prkc activity by reducing DAG/Ca2+ so as to inhibit the occurrence of a series of cascade reactions such as the regulation of ERK signaling pathway and finally increase the expression of Mapt, resulting in antidepressant effect, while the specific upstream and downstream relations of the pathways still need further study.

Monoamine hypothesis is a major topic in depression research. It suggests that an imbalance in the monoamine neurotransmitters such as DA is the cause of depression [51]. Studies showed that, compared with the depression model group, acupuncture could increase DA level [52, 53]. Recent studies have revealed that DAT play an important role in depression and DAT block presented potential antidepressant effect [54]. DAT located at the membrane of presynaptic terminals plays an important role in regulating the DA intensity and the duration of dopaminergic neurotransmission in the synaptic cleft by removing DA from the synaptic cleft into presynaptic neuron [55]. Interestingly, a lower availability of DAT has been reported in anhedonic depressed patients, suggesting a possible role of DAT as specific therapeutic target in patients with anhedonia [56]. Although the present results of Western blot verification are inconsistent with iTARQ in our research, most studies still support the high DAT expression level in depression [57]. In depressive state, DAT expression increased, and the intercellular DA would be absorbed by cells containing DAT, resulting in intercellular DA reduction. And EA may achieve antidepressant effect by reducing DAT expression; avoiding DA was taken by cells containing DAT.

It has been proposed that alterations in the activity of Th in the central noradrenergic system may lead to depression [58]. Antidepressant Fluoxetine could reverse depressive symptoms by increasing plasma catecholamine levels via increasing Th in CUMS rats [59]. Another study found essential oil from* Asarum heterotropoides* could effectively inhibit depression-like behavioral responses by increasing brain Th expression in mice challenged with stress [60]. The two findings are consistent with our iTRAQ results. It indicates that Th may be a biological target or mechanistic rationale in the development of alternative medical treatments for depression [61]. On the contrary, a study found Th expression was elevated following exposure to chronic stress in the locus coeruleus (LC) [62].

There was not much literature focusing on the relationship between Th and depression. One study of EA on Parkinson's disease (PD) found that EA had a minimal impact on the Th-positive profiles of the ipsilateral ventral tegmental area [63]. Although the change did not have statistically significant difference in the present study, Western bolt showed the tendency that expression of Th was decreased in the depressed state and could be increased by EA intervention in the hippocampus.

Pathway analysis found that DAT and Th were involved in dopaminergic synapse signaling pathway. The decreasing in Th expression could facilitate the conversion of L-lysine to dihydroxyphenylalanine (dopa). Dopa is the precursor of DA, which affects the production of DA. On the other side, once the DAT expression increased, the synaptic cleft DA would be taken into the synaptic neuron by DAT [64]. The low level of DA in synaptic cleft would result in anhedonia.

The precise signaling mechanism regulated by EA has not been worked out. Taken together the results of iTRAQ and Western blot, we speculated that EA may regulate the dopaminergic synapse signaling pathway. A possible mechanism is that EA increases Th expression and decreases DAT expression, and upon neuronal excitation (action potential) DA is released into the synaptic cleft in brain. DA effects the D2 receptors that can activate reward system. And then, the reward system gives positive reinforcement to antidepressant.

In the DAT and Th expression change, the results of Western blot are inconsistent with the results of previous iTRAQ proteomics. The reasons are probably as follows.

Firstly, the distribution and the concentration of DAT and Th are different in different brain regions and may vary with time or other factors.

Secondly, to some extent, the sample size for quantitative study is small, and there was individual difference.

Thirdly, the dopaminergic regulation may be different from individual to individual in depressed subjects, and DAT levels can be reduced only in certain patients according to the specific clinical profile [65].

Although Th and DAT are not significantly different in Western blot validation, the involvement of brain dopaminergic system including Th and DAT has been noted in major depressive patients [66, 67]. The role of EA on its regulation cannot be ignored.

## 5. Conclusions

In conclusion, based on iTRAQ technology taken into full consideration of whole proteins regulated by EA in depression, differential proteins were screened and verified by using Western blot. Reinforcing the notion that these are important targets for EA activity, our study provides the clear and novel evidence for the participation of Prkc and Mapt in the antidepressant-like effect of EA in CRS rats. DAT and Th are two novel targets of depression pathophysiology that deserved more research. Therefore, the results suggest that EA should be further investigated as a potential antidepressant treatment. In addition, not only single indicator but also the MAPK and dopaminergic synapse signaling pathways warrant further study, indicating that EA plays an antidepressant effect via regulating different pathways rather than single pathway. Our study provides clues and evidence for the treatment of depressive patients who are not responding to current antidepressant drugs. In future clinical studies, proteins in peripheral blood can be verified by serum or plasma, so as to find effective targets for EA on depression from the central to peripheral systems.

## Figures and Tables

**Figure 2:**
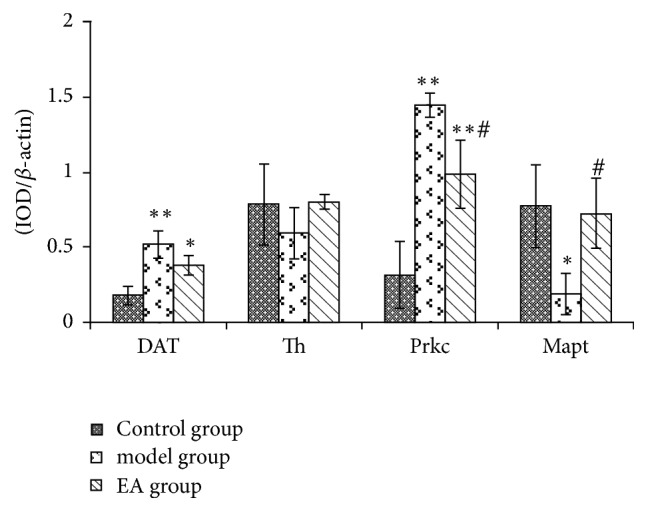
Candidate proteins expression change in three groups. IOD: the integrated optical density; ^*∗*^*p* < 0.05, ^*∗∗*^*p* < 0.01 as compared with control group; ^#^*p* < 0.05, as compared with model group.

**Figure 3:**
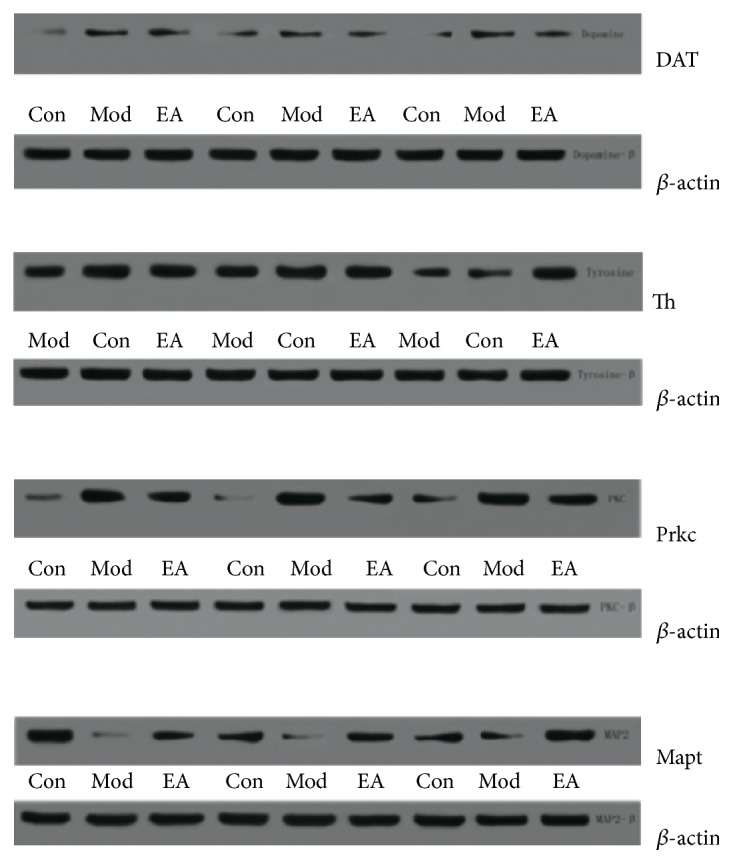
Representative Western blots showing levels of DAT, Th, Prkc, and Mapt in the hippocampus of the following groups (*n* = 4 per group, repeated 3 times): control, model, and EA.
